# Graphene hybrids: synthesis strategies and applications in sensors and sensitized solar cells

**DOI:** 10.3389/fchem.2015.00038

**Published:** 2015-06-30

**Authors:** Sushmee Badhulika, Trupti Terse-Thakoor, Claudia Villarreal, Ashok Mulchandani

**Affiliations:** ^1^Department of Electrical Engineering, Indian Institute of TechnologyHyderabad, India; ^2^Department of Bioengineering, University of CaliforniaRiverside, CA, USA; ^3^Department of Material Science and Engineering, University of CaliforniaRiverside, CA, USA; ^4^Department of Chemical and Environmental Engineering, University of CaliforniaRiverside, CA, USA

**Keywords:** graphene hybrids, graphene-CNTs, graphene-QDs, sensors, energy conversion

## Abstract

Graphene exhibits unique 2-D structural, chemical, and electronic properties that lead to its many potential applications. In order to expand the scope of its usage, graphene hybrids which combine the synergetic properties of graphene along with metals/metal oxides and other nanostructured materials have been synthesized and are a widely emerging field of research. This review presents an overview of the recent progress made in the field of graphene hybrid architectures with a focus on the synthesis of graphene-carbon nanotube (G-CNT), graphene-semiconductor nanomaterial (G-SNM), and graphene-metal nanomaterial (G-MNM) hybrids. It attempts to identify the bottlenecks involved and outlines future directions for development and comprehensively summarizes their applications in the field of sensing and sensitized solar cells.

## Introduction

The recent emergence of graphene, a one atom thick sp^2^-hybridized two-dimensional (2D) honeycomb lattice of a carbon allotrope, has opened up innumerable opportunities in the field of materials science and nanotechnology research. Graphene exhibits extraordinary mechanical, thermal, and electronic properties such as high specific surface area per unit volume, high chemical stability, excellent thermal conductivity (up to 5000 W m^−1^ K^−1^) (Balandin, 2011) and a unique band structure with band-tuning ability and extremely high carrier mobility (in excess of 1,00,000 cm^2^/V· s) (Novoselov et al., 2004, 2005; Zhang et al., 2005; Bolotin et al., 2008). Thus, it is envisioned as a superior alternative to silicon and is widely used in applications such as solar cells, field-effect transistors, batteries, super capacitors (Wang et al., 2008b; Yoo et al., 2008), chemical sensors, and biosensors (Huang et al., 2011; Avouris and Dimitrakopoulos, 2012; Shen et al., 2012).

However, these properties only emerge in the 2D planar direction of the graphene structure, limiting its scope and application. New efforts in graphene research have attempted to address this weakness by developing structures wherein graphene acts as a platform for support, scaffold, or a 2D planar substrate for anchoring other nanomaterials. For example, carbon nanotubes (CNTs), whose properties emerge in the axial direction, can be functionalized onto the surface of graphene, combining the properties of the two carbon allotropes in all directions while allowing for an increased active surface area and faster electron transfer kinetics. Similarly, combining metal/metal oxide nanoparticles (NPs) with graphene overcomes NP shortcomings of low stability and tendency to aggregate resulting in highly stable electrochemical sensing platforms for anchoring with good dispersion, excellent conductivity and catalytic properties of NPs. This transformation of 2D graphene into three-dimensional (3D) architectures expands the functionality of nanomaterials and serves as novel hybrid electrode material for applications in electronics (Kim et al., 2014), energy storage (Prasad et al., 2014), and sensing (Dong et al., 2012; Hwa and Subramani, 2014).

In this review we discuss different aspects relevant to nanoscale architecture of graphene-carbon nanotube (G-CNT), graphene-semiconductor nanomaterial (G-SNM), and graphene-metal nanomaterials (G-MNM) hybrid nanostructures based on graphene as a multifunctional platform. Nanoscale architecture is key for the performance of these novel materials, aspects such as size, geometry, chemistry of the building blocks, relative orientation of crystals, as well as the limitations and fabrication methods must be considered during development of devices that make use of graphene hybrids. Given their wide scope, we have briefly reviewed the applications of these graphene hybrids primarily in two of the most advanced fields, namely sensing and energy harvesting. This review shall enable the reader to realize the versatility graphene hybrid materials offer by virtue of their synergistic properties. Wherever applicable, the limitations of the present approaches and future research directions have been highlighted.

## Synthesis strategies for graphene hybrids

Hybridization of graphene variants such as epitaxial graphene, graphene oxide (GO) or chemical vapor deposition (CVD) graphene, with CNTs, semiconducting nanomaterials (SNMs), and metal nanomaterials (MNM) can be done by two fundamentally different approaches. The **assembly method** synthesizes graphene and CNTs, SNMs, or MNMs separately and then assembles them together. The ***in situ* method** grows CNTs, SNMs, or MNPs directly on the graphene structures.

### Assembly method

This is a widely used method in graphene hybrid synthesis involving either self- or chemical assembly. Self-assembly is a technique based on the spontaneous organization that the components (CNTs, SNMs, or MNPs and graphene/GO) undergo when encountered in a liquid media through simple physical dispersion. Chemical assembly uses a chemical linker or electrostatic interactions to assemble the components in a layer-by-layer-method to form lamellar architecture.

#### Assembly of graphene and CNTs

Graphene and CNTs interact via van der Waals forces or π –π interaction through their aromatic sp^2^ structure to form a high surface area hybrid structure. Electrostatic or covalent assembly is carried out using highly oxygenated functional groups on GO or by introducing complementary functional groups in the sp^2^ lattice of CNTs and graphene through different chemistries.

##### Physical mixing/dispersion method

GO prepared by Hummer's method has many oxygen containing functional groups and is therefore readily soluble in water. CNTs, however, tend to agglomerate in while suspended in water and surfactants are needed for uniform dispersion. The high dispersibility of GO in water and its π –π interaction with CNTs facilitates the dispersion of CNTs without use of surfactant. Additionally, the CNTs help prevent the restacking of GO sheet (Qiu et al., 2010; Chen et al., 2012).

**Figure 1:**
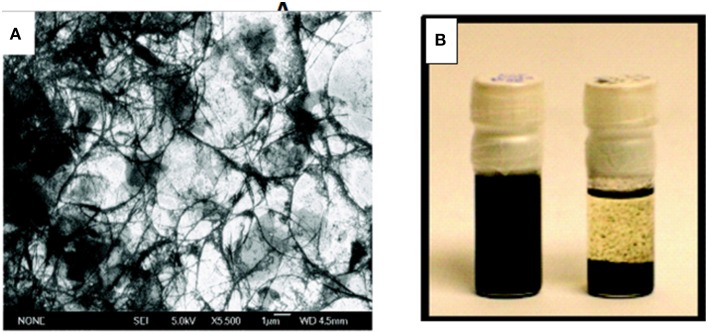
**Preparation of rGO-CNT hybrid by dispersion and reduction of GO-CNT mixture in anhydrous hydrazine. (A)** SEM image of GO-CNT, **(B)** Stable dispersion of GO-CNT in anhdrous hydrazine (left) compared to organic solvent (right) after 12 h. [Reprinted with permission from Tung et al. (2009). Copyright (2009) American Chemical Society].

**Figure 2:**
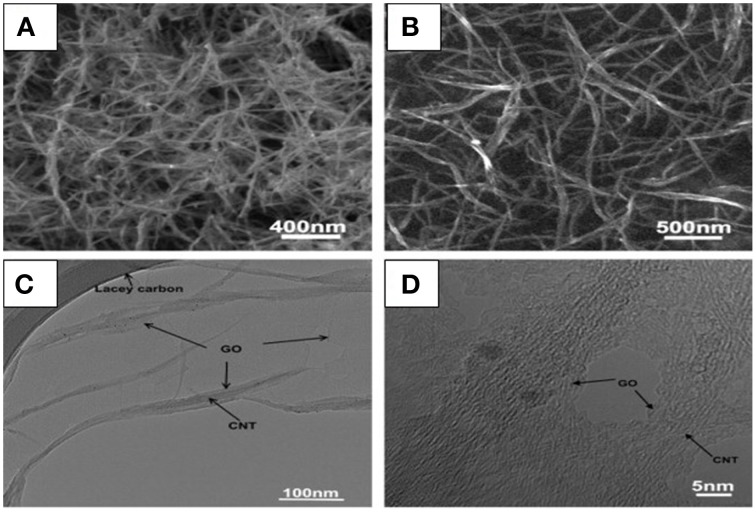
**Core shell G-CNT hybrid structure. (A)** SEM image of SWCNTs, **(B)** SEM image of SWCNT/GO, **(C)** TEM image of SWCNT/GO, **(D)** High-resolution TEM image of SWCNT/GO (Dong et al., 2011b). [Reprinted with permission from Dong et al. (2011b). Copyright (2011) Elsevier].

Although increasing the number of oxygen containing groups improves the aqueous dispersibility of GO, they also increase the defects in the sp^2^ lattice structure increasing sp^3^ content thus making GO sheets electrically insulating. The conductivity of GO can be restored by reduction or deoxygenation using chemical and hydrothermal methods. (Gao et al., 2009; Shin et al., 2009; Pei et al., 2010). Reduction of GO after or simultaneously during GO-CNT hybrid assembly results in a conductive single layer of chemically transformed graphene with uniformly distributed network of CNTs. Such a hybrid was prepared by sonication assisted mixing of acid functionalized CNTs with GO and simultaneous deoxygenation under alkaline condition (Chen et al., 2012). A similar GO-CNT hybrid was prepared by dispersing the powder forms of GO and slightly oxidized CNTs in anhydrous hydrazine (Tung et al., 2009). The same study reported anhydrous hydrazine assisted dispersion of GO-CNT to be more stable in aqueous than organic solvents (Figure 1). Core shell structure of GO and CNTs (a CNT wrapped by multiple GO sheets) were formed when nanosized GO sheets were dispersed with CNTs (Figure 2) whereas micron sized GO sheets prefer adhesion of multiple CNTs onto the surface of a single GO sheet (Dong et al., 2011b).

##### Chemical/layer-by-layer (LBL) assembly

**Figure 3:**
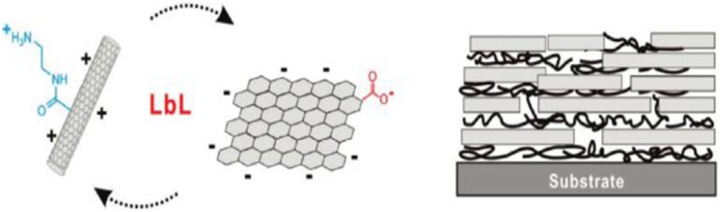
**Schematics of Layer by Layer assembly of rGO-CNT**. [Reprinted with permission from Hong et al. (2010a). Copyright (2010) American Chemical Society].

Functionalized graphene and CNTs are assembled based on their respective functionalities or by simple solvent wetting method. (Hong et al., 2010a) reported the layer-by-layer assembly of positively charged multi-walled carbon nanotubes (MWCNT) between oppositely charged reduced GO sheets (rGO) through electrostatic interaction. Amino groups were introduced on acid functionalized MWCNT using excess ethylenediamine mediated EDC chemistry. By repeated spin coating of NH_2_-MWCNT and rGO successively on the substrate, a multilayer hybrid film was formed. The bridging of the graphene sheets by CNTs improved the overall conductivity and mechanical flexibility of the hybrid structure as shown in the schematics in Figure 3.

GO and CNTs were chemically bonded to form a lamellar structure using an amidation chemistry (Jung et al., 2013). Carboxyl groups on GO were activated using NHS/EDC chemistry to form NHS-GO. Amine functionalized CNTs were synthesized similarly then attached between graphene sheets acting as a spacer to improve the electrolyte diffusion as well as forming 3D conducting network.

In another assembly method, known as solid phase stacking approach (Li et al., 2010a), graphene was grown on Cu using CVD method. A CNTs film was grown separately on Ni mesh using CVD, then separated and stacked on the graphene/copper foil. To improve the adhesion between graphene and CNTs, a drop of ethanol was placed on the assembly and the copper foil was etched away. The resultant hybrid had good mechanical strength and transparency needed for optical electrodes.

Though the assembly based methods resulted in a high surface area 3D conductive network of graphene and CNTs, they had issues of multiple processing steps, no close interaction of CNTs with graphene, and difficulty in controlling density/layers of graphene sheets in GO because of its tendency to agglomerate in the hybrid structure, thereby affecting the available surface area and overall conductivity. These limitations have been addressed by growing CNTs *in situ* on graphene or by growing graphene and CNTs simultaneously.

#### Assembly of fully-grown SNM and graphene

The simplest methods involve application of a layer of SNM dispersion/suspension as a coating on graphene by spreading, doctor blading, spraying, spinning, dipping, etc. and then drying to remove the solvent. In drop-casting, the suspension of SNMs is dropped onto the graphene and the solvent is allowed to evaporate. Drop casting of ZnO resulted in high density conglomeration of nanoparticles on rGO (Watanabe et al., 2014).

SNMs and graphene can be mixed in a liquid through mechanical stirring, bubbling, or sonication and induced into assembly by different interactions between the surfaces. GO is preferably used in aqueous reaction due to its hydrophilic nature compared to other forms of graphene. Yang et al. (2010) prepared the hybrid of TiO_2_/rGO by dispersing a commercial paste of TiO_2_ NP in GO aqueous solution, using an binding additive, stirring, sonicating for 30 min, and then applying the resulting paste on fluorine doped tin oxide (FTO) with doctor-blade. The dried electrode was then treated with hydrazine vapor and annealed to completely reduce the GO.

**Figure 4:**
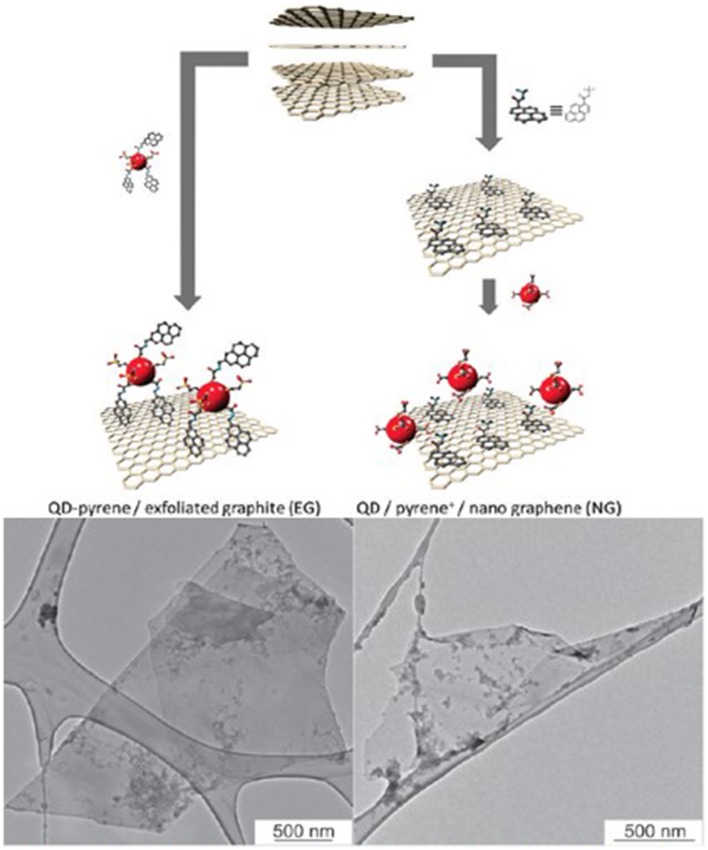
**Schematic representation of functionalized QDs stacked on exfoliated graphene and nanographene stacked with pyrene and functionalized with QDs, and their TEM images, on the left and on the right, respectively**. [Reprinted with permission from Katsukis et al. (2012). Copyright (2012) American Chemical Society].

Intermolecular non-covalent forces, such as hydrophobic–hydrophobic/π –π interactions, between graphene and molecules with similar structure, like pyrene, can be used to graft semiconductor nanoparticles on graphene in suspension. Katsukis et al. (2012) employed two different strategies for immobilization of water-soluble CdTe QDs on graphene. In one, the QDs were covalently functionalized with pyrene by organic reactions and then stacked on exfoliated graphite. In the second, nanographene was first stacked with positively charged pyrene by sonication, and then negatively charged QDs were immobilized on top (Figure 4). The electronic coupling of the materials was better in the latter, due to larger exfoliation of the graphite.

**Figure 5:**
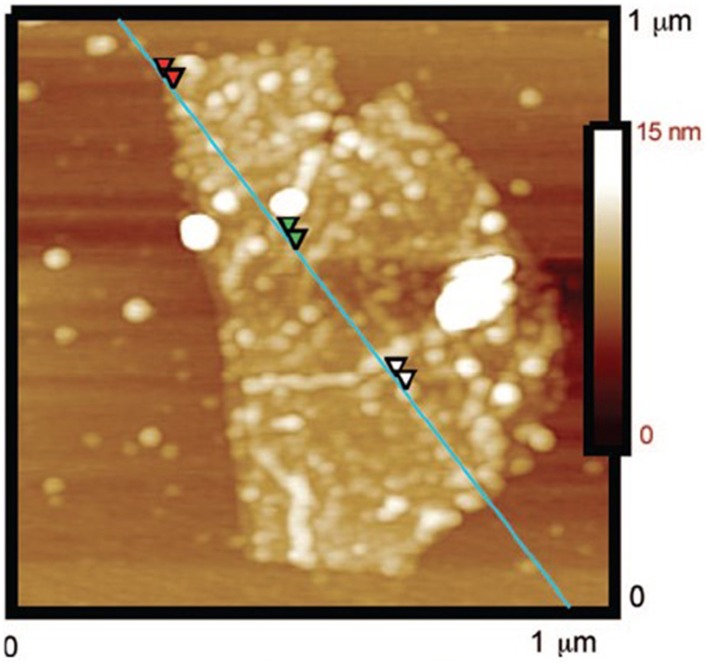
**AFM image of TiO_2_-GO hybrid synthesized by photochemical reduction**. [Adapted with permission from Williams et al. (2008). Copyright (2008) American Chemical Society].

Certain inherent properties of SNM and graphene are useful during the formation of hybrids. For example, the excellent photocatalytic activity of TiO_2_ has been used to mediate the photochemical reduction of GO with simultaneous assembly of TiO_2_ NPs onto the surface of rGO, in a single step method that is environmentally friendly (Williams et al., 2008). TiO_2_, a mild reductant, doesn't modify the sp^2^ hybridized carbon. At the same time, the collapse of rGO nanosheets is prevented by the interaction of TiO_2_ NP with rGO, through physisorption, electrostatic binding, or charge transfer resulting in hybrid nanosheets (Figure 5). Pengzhan et al. (2013) used both self-assembly and layer-by-layer method for the hybridization of monolayer titania, exfoliated from titanates, and GO. These lamellar materials synthesized by layer-by-layer methods are of importance for architectures involving new 2D materials, such as BN, MoS_2_, Bi_2_Te_3_, MnO_2_ etc.

#### Assembly of MNMs and graphene

**Figure 6:**
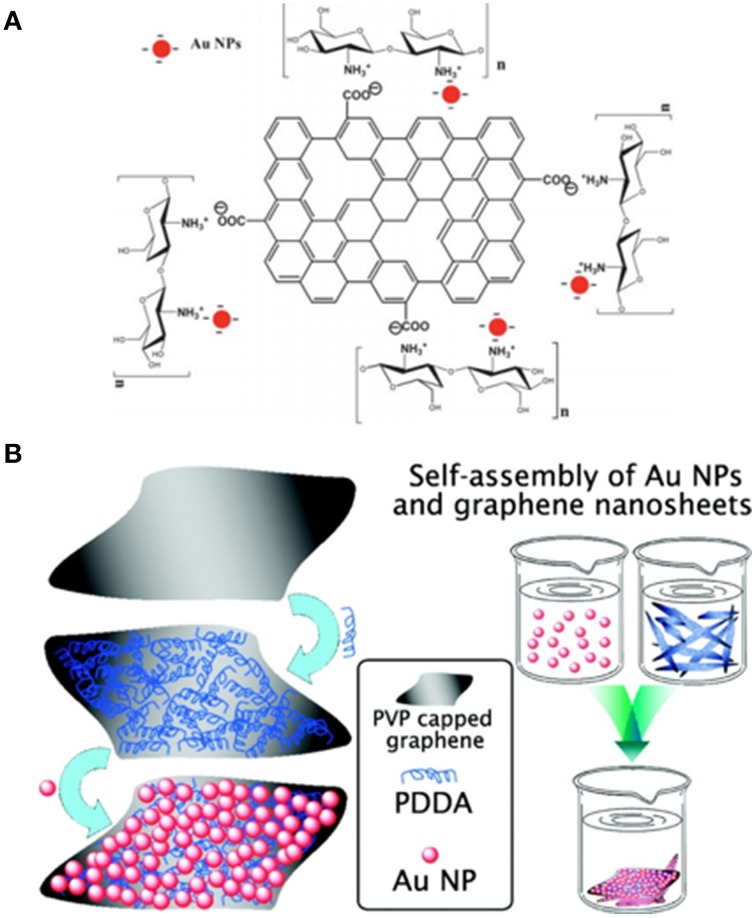
**Self-assembly of already synthesized AuNP on (A) RGO/chitosan (B) on polyelectrolyte poly(diallyldimethyl ammonium chloride) (PDDA) functionalized graphene nanosheets (GNs)**. [Reprinted with permission from Chang et al. (2011); The Royal Society of Chemistry and Fang et al. (2010). Copyright (2010) American Chemical Society].

MNMs are generally synthesized through the conventional chemical reduction of metal salts. This method results in a negatively charged surface which can be used as is, after further chemical modification for self-assembly (Chang et al., 2011; Li et al., 2013b), or through a chemical linker such as pyrene butanoic acid (Hong et al., 2010b), polycationic protamine (Fu et al., 2012) or poly polyelectrolyte poly(diallyldimethyl ammonium chloride) (PDDA) (Fang et al., 2010). Li et al. (2013b) synthesized graphene metal nanoparticle (MNP) hybrid by electrostatic self-assembly of negatively charged sulfonated graphene nanosheets and positively charged gold nanoparticles (AuNP). Figure 6 shows the schematics of assembled graphene-MNP hybrid.

### *In-situ* growth method

Assembly processes are generally simpler than *in situ* fabrication, but produce lower quality materials with undesired phenomenon like aggregation, poor contact, residues, and non-uniformity of the films. On the other hand *in situ* growth of graphene hybrids provides better fabrication control in terms of morphology, density, and orientation of the hybrid structures with ohmic contact of CNTs or SNMs with graphene resulting in high conductivity architectures.

#### *In situ* growth of G-CNT hybrid

This involves the direct growth of graphene and/or CNTs using CVD to ensure Ohmic contact between graphene and CNTs. It also gives more control over the morphology of the carbon nanostructure by tuning the growth conditions. Compared to assembled hybrids, *in situ* methods involve less processing steps and guarantee more uniform distributed growth of CNTs on the graphene. *In situ* G-CNT hybrids can be further classified as one-step and two-step hybrids based on the number of CVD steps involved.

##### One-step G-CNT hybrids

Growth of graphene and CNTs is carried out simultaneously in a single CVD step therefore these hybrids are termed here as one-step hybrids. In this method, catalysts for CNTs growth, such as Fe and Ni, are deposited onto the growth substrate of graphene such as copper foil (Paul et al., 2010; Ghazinejad et al., 2013), nickel foam (Xiaochen et al., 2012), and MgO (Zhu et al., 2012b) by thin film evaporation methods or by immersing in metal salt solution. The catalyst coated substrate is subjected to CVD growth using a carbon source gas for simultaneous growth of graphene and CNTs. Lamella-like mixed catalyst, Fe/MgO and MgO were prepared using the hydrothermal method and subjected to CVD for the growth of G-CNT hybrid (Zhu et al., 2012b). MgO catalyzed the growth of graphene and the Fe/MgO layer catalyzed growth of CNTs.

Copper alone can act as catalyst for graphene and CNT growth depending on its physical and chemical state. Coating of Si-NP on the copper foil resulted in growth of G-CNT in one-step (Dong et al., 2011a). In this method, Si-NP acts as the scaffold onto which copper at high temperature gets deposited and forms the catalyst nanoparticles promoting CNT growth. The bamboo-like morphology of CNTs was obtained with uniform coverage on graphene.

**Figure 7:**
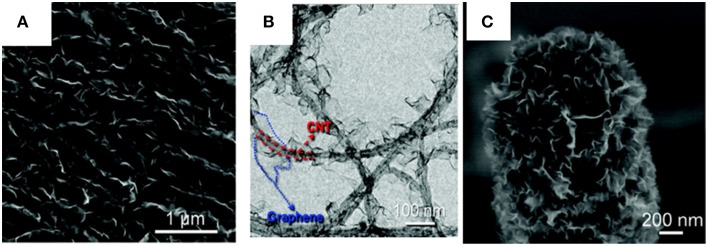
**Few layer graphene leafy structure grown on CNT (CNT-FLG) (A) SEM image CNT-FLG hybrid of (B) TEM image CNT-FLG hybrid (C) closer view of tip of CNT-FLG hybrid**. [Reprinted with permission from Yu et al. (2011). Copyright (2011) American Chemical Society].

A chemically fused and catalyst free G-CNT hybrid was grown using CNTs as the substrate for graphene growth (Yu et al., 2011). Here, CNTs are subjected to plasma enhanced chemical vapor deposition (PECVD) with argon as a carrier gas and methane as a carbon gas. Argon plasma generates defects in the CNTs forming nucleation sites and cracks methane into free carbon, resulting in few-layer leafy graphene structures (Figure 7).

It has been reported that the electronic and thermal conductivities of the CNTs are significantly reduced in forest-like or entangled structure due to discontinuous contact of CNTs to each other (Wang et al., 2008a). To address this issue, the growth of vertical and inverted CNT structures on the graphene was carried out using a one-step method. Das et al. (2011) synthesized graphene along with inverted CNTs using a thin film of iron catalyst (20 nm) on the Si substrate without any additional catalyst. On the other hand, Du et al. (2011a) carried out the growth of vertically aligned CNTs in between the thermally expanded highly ordered pyrolytic graphite (HOPG) using pyrolysis of catalyst source. In this hybrid, HOPG were treated with acids which caused acid molecules to be physically trapped in the HOPG sheets. Next, heating the HOPG sheets caused them to separate. Separated HOPG sheets were chemically coated with SiO_2_ and then vertically aligned CNTs (VACNTs) were grown between the sheets using thermal pyrolysis of iron containing precursor to from a 3D pillared structure. 3D pillared structures made of CNTs in between parallel graphene sheets possess desirable out-of plane electronic transport and superior mechanical properties. They retain the intrinsic properties of the individual components and have improved surface area for applications such as immobilization of materials and electrolyte exchange through diffusion (Wang et al., 2014).

##### Two-step G-CNT hybrids

Depending on the metal catalyst (CNT growth) and metal substrate (graphene growth) interaction, it may be necessary to use a two-step growth method. Specifically in the case of iron catalyst on copper foil it has been reported that CNT growth was difficult due to hindrance of the active catalyst surface by possible formation of Fe/Cu alloy (Duc Dung et al., 2012). Generally, to grow CNTs, a thin metal film is first deposited on the substrate which forms active catalyst NPs at high temperature. NP formation can be controlled by the thickness of the metal film and metal's affinity/contact angle to the substrate. When carbon source gas is passed, it gets cracked at catalyst NP to form carbon and hydrogen. Once carbon saturates the NPs it precipitates out to form CNTs. Graphene facilitates the formation of active catalyst NPs by acting as a barrier to prevent the formation of Fe/Cu alloy. Two step hybrids with single to few walled CNTs have been shown to seamlessly connect with graphene (Zhu et al., 2012c). In this method first Fe film catalyst (1 nm) and then alumina layer (3 nm) was deposited on the CVD grown graphene/copper foil. The metal coated graphene/copper foil was again subjected to CVD for CNT growth. Alumina layer acted as the floating buffer layer which helped in covalent binding of CNTs to graphene as well as in controlling the diameter of CNTs.

Free standing, flexible and highly conductive G-CNT nanocomposite was grown using CVD method with cobalt functionalized chemically reduced graphene sheets on a polymeric membrane (Su et al., 2010).

#### *In situ* growth of graphene-SNM hybrid

Graphene has proven to catalyze the growth of semiconductor materials, in some cases acting to assist in fabrication control, regulating density, and orientation. For *in situ* fabrication, the interface structure will largely depend on the growth mechanism of the SNM on the graphene substrate. For example, in-plane defects on graphene provide preferential sites for nucleation and clustering of deposited gold, used for ZnO-growth catalysis (Biroju et al., 2014).

We have categorized the most common techniques for *in situ* synthesis of SNM/graphene hybrid according to the phase in which the growth occurs, gas or liquid, due to the fundamental contrast between the two in terms of growth mechanisms and techniques.

##### Gas phase processes

These include physical evaporation deposition (PVD), CVD, metal-organic vapor phase epitaxy (MOVPE), and atomic layer deposition (ALD) for the growth of SNM/graphene hybrids.

CVD is one of the most common methods for ZnO growth on graphene, given the relatively low melting and boiling point temperature of Zn (Biroju et al., 2014). A characteristic feature of this method is the range of morphologies that can be obtained, ranging from nanometric walls, particles, rods, tetrapods, ribbons, and beyond by varying the pretreatments done to graphene and the use of different metallic catalysts. For example, using Au as catalyst on the graphene surface will yield aligned nanorods while an absence of Au yields randomly oriented nanoribbons. A vapor-solid mechanism is suggested, in which the large quantity of Zn migrates on the surface of graphene with less sticking coefficient and eventually leads to random orientation of nanoribbons.

ZnO has also been grown on graphene using metal-organic vapor-phase epitaxy (MOVPE) by flowing oxygen and an organic Zn precursor in a gas phase reactor. The gas molecules break up when in contact with graphene surface and ZnO is immediately adsorbed onto the surface. This method results in highly crystalline material without defects such as stacking faults or dislocations (Kim et al., 2009).

Titania, on the other hand, is preferably deposited by other methods due to the high transition temperatures of titanium. For example, TiO_2_ nanoparticles in either anatase or rutile phase with less than 5 nm diameter have been deposited on graphene by direct e-beam evaporation (Wang et al., 2011b).

ALD has also been used for TiO_2_ growth, by a gas-solid synthesis route featured by two sequentially cyclic self-limiting half-reactions that result in a layer-by-layer growth mode. Unlike CVD, in ALD, the precursors are not decomposed by themselves under the reaction conditions, but make direct reaction in the substrate. A graphene nanosheet has been coated with TiO_2_ using ALD method at temperatures as low as 150 to 250°C (Meng et al., 2011).

##### Liquid phase processes

These include traditional solution chemistry, electrochemical, hydro/solvothermal methods and more recently developed techniques like electrospinning and successive ionic layer adsorption and reaction (SILAR).

Yan et al. (2010) synthesized graphene quantum dots through stepwise solution chemistry and functionalized them covalently at the edges with 1,3,5-trialkyl phenyl to increase their solubility. Phenyl groups were attached to one of the intermediate molecules instead of the final graphene. The flexible peripheral phenyl chains bend and enclose the graphene in all three dimensions, preventing agglomeration. Because of the good affinity of the side chains with solvents, the inter-graphene attraction is overcome, and graphene QDs are entropically pushed apart, resulting in good solubility in common solvents.

**Figure 8:**
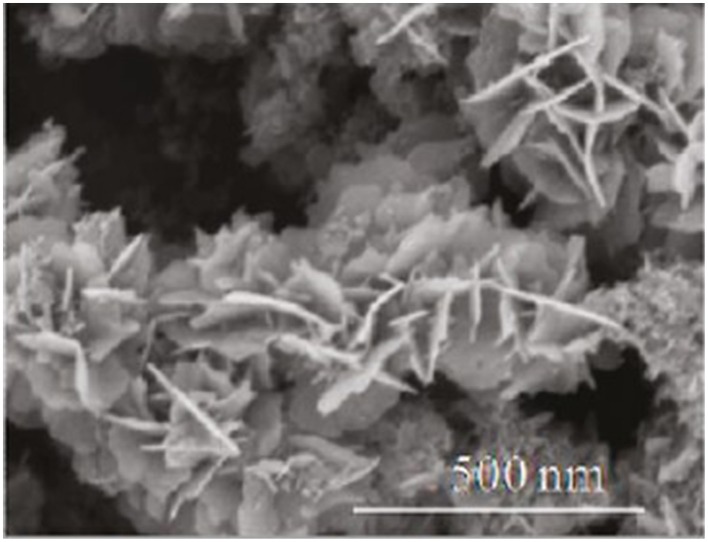
**SEM image Cu_2_S/rGO composite grown *in situ* through solution chemistry**. [Reprinted with permission from Radich et al. (2011). Copyright (2011) American Chemical Society].

It takes several steps of solution chemistry to obtain the *in situ* Cu_2_S/rGO hybrid (Radich et al., 2011). First, GO sheets were exfoliated by sonication and complexed with Cu^+^ by electrostatic interactions of the cations with the highly electronegative oxygen moieties in GO. Ethanol was added and the Cu^+^ disproportionation reaction occurs, partly aimed by sonolytic reduction and the GO acting as nucleation sites for Cu^0^. The GO/Cu hybrid was spin coated on an FTO glass and GO was reduced under UV irradiation or by annealing at 250°C. The material was then immersed in a polysulfide solution and the Cu on rGO converted to Cu_2_S. Figure 8 shows the SEM image of the Cu_2_S/rGO composite.

Hydrothermal and solvothermal methods are largely used and preferred over gas phase methods when using polymeric substrates for flexible products, given the lower temperatures that are required. The difference between the two methods lies in the solvent used, water or organic. For ZnO in hydrothermal methods, no preferential growth was observed in step edges and domain boundaries possibly because the particles nucleate and grow in the solution rather than on the surface, so the surface state doesn't influence nucleation as strongly as in the gas phase. The effects of temperature, pH, and concentration are similar using either exfoliated or CVD graphene (Yong-Jin et al., 2011; Liu et al., 2013). Organic solvents, such as benzyl alcohol:ethanol, used in solvothermal synthesis, is preferred when the precursor is an organic molecule, for example for TiO_2_, titanium isopropoxide is used as precursor, and the molecules are grafted on the graphene by chemisorption. TiO_2_ nanoparticles grown by solvothermal showed an increase in size with growth temperature raise (He et al., 2013).

TiO_2_ nanotubes on carbon nanosheets derived from GO have also been synthesized by sequential combination of solvo/hydrothermal methods (Peng et al., 2010). First, metallic Ti was intercalated with GO by mixing a solution of titanium tetraisopropoxide and a GO suspension and refluxing at 65°C. Then GO/Ti was mixed with 10 M NaOH and treated for 24 h at 150°C. These 1D TiO_2_ structures deposited two-dimensionally along the surface of carbon nanosheets and never aggregated.

Successive ionic layer adsorption and reaction method (SILAR) is a technique largely used for QD synthesis in variety of substrates. It has been combined with layer-by-layer to fabricate a stacked triple-composite with titania nanosheets (TNS), rGO, and QD (CdS). After one cycle of layer-by-layer stacking of TNS and GO, the material was subjected to the SILAR cycle, a successive immersion in solutions of Cd(NO_3_)_2_, H_2_O, Na_2_S, and H_2_O, providing the ions for the CdS. The described stacking was repeated several times. Finally, the GO was reduced with hydrazine evaporation and annealed at 400°C (Yang et al., 2012; Wang et al., 2013b).

##### Electrospinning

Electrospinning is a simple and cost-effective technique to fabricate 1D nanostructures. It has been used to fabricate TiO_2_-graphene composite conductive nanofiber mats. The process involves mixing the carrier solution and graphene powder and feeding the mixture into an electrospinning setup, at a prescribed temperature and applied potential. The applied voltage and very slow deposition rate allows the formation of nanofibers (Madhavan et al., 2012). Same technique was used by Zhu et al. (2012a), however, theirs involved the use of exfoliated graphene sheets functionalized with cetyltrimethylammonium bromide to make them soluble in DMF. A well-connected hybrid structure with randomly distributed rice grain-shaped 1D TiO_2_ structures on graphene sheets was obtained.

#### *In situ* growth of graphene-MNM hybrid

**Figure 9:**
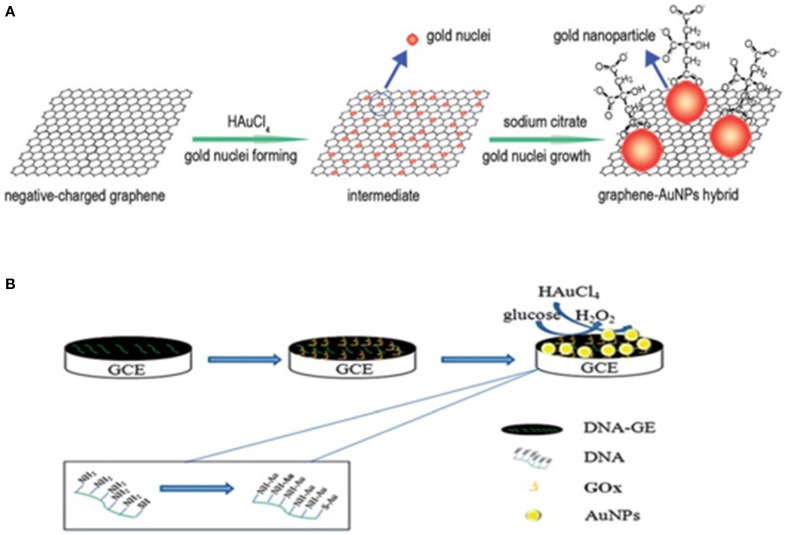
***In situ* grown graphene-AuNP hybrid**. Schematics of synthesis of graphene-AuNP hybrid **(A)** using chemical and **(B)** biocatalytic reduction method. [Reprinted with permission from Chen et al. (2011b) and Zheng et al. (2011); The Royal Society of Chemistry].

*In situ* chemical (Dong et al., 2010b; Li et al., 2010b; Qiu et al., 2011), electrochemical (Hu et al., 2010, 2011; Gao et al., 2011; Liu et al., 2011) or thermal (Yoo et al., 2009) reduction of metals (Au, Pt, Pd, Ru, or bimetallic etc.) and precursor salts suspended with graphene, is the most common and widely used strategy for G-MNS hybrid synthesis. Biocatalytic reduction has also been reported where enzyme glucose oxidase coated graphene electrode catalyzes the oxidation of glucose producing H_2_O_2_ which in turn reduces the Au precursor salt (HAuCl4) and produces AuNP on graphene (Zhang et al., 2011; Zheng et al., 2011). Figure 9 shows the schematics of *in situ* growth G-MNP hybrids. Other methods such as microwave assisted one pot synthesis for Pt/Ru NP on graphene (Wang et al., 2011c) and radio-frequency catalytic CVD for single step synthesis of graphene–Au and –Ag hybrid nanostructures has also been reported (Pruneanu et al., 2013). Chen et al. (2011a) have shown that by taking advantage of redox reaction between GO and PdCl_4_^2−^, PdNP-GO hybrid can be synthesized without use of any external reductant.

## Applications

### Sensors

#### Graphene hybrids as electrochemical sensors

Glucose biosensors are one of the primary areas of interest in the field of clinical biosensing due to an increasing number of diabetic patients worldwide and dominate more than 90% of the total world biosensor market. Most of them are enzyme based amperometric detection biosensors with recent reports of non-enzymatic glucose detection as well. Platinum nanoflowers decorated on *in situ* grown G-CNT electrodes (Badhulika et al., 2014) and GO electrode (Wu et al., 2013a) were reported for non-enzymatic detection of blood glucose.

Enzymatic glucose biosensors primarily use glucose oxidase (GOx) which is stable and highly specific for glucose. Glucose oxidase catalyzes the oxidation of the glucose in the presence of oxygen to produce gluconolactone and hydrogen peroxide. Flavin adenine nucleotide (FAD) is the active center of the enzyme responsible for the redox reaction. First generation glucose biosensors electrochemically detect H_2_O_2_ generated at the electrode due to enzymatic action but generation of H_2_O_2_ is oxygen dependent and requires higher detection potential at which other redox compounds interfere. Second generation biosensors completely eliminate the oxygen dependency in hydrogen peroxide formation using redox mediators. These redox mediators are mostly in free form and can diffuse freely to reach FAD or wired to an enzyme transfer electrons to the electrode and vice versa, subsequently generating an amperometric signal. Third generation glucose biosensors work at lower potential without using any redox mediators by direct electron transfer (DET) from redox center to the electrode.

DET is difficult to achieve with GOx enzyme on bare electrodes such as a glassy carbon electrode, as FAD is deeply embedded in the structure. Graphene based hybrids provide an efficient and highly conductive matrix for enzyme immobilization and faster electron transfer in achieving the DET for GOx without use of any mediator thus finding application in glucose sensing. Though exact mechanism by which theses hybrids achieve DET is unknown, proximity of highly conductive graphene and its 3D hybrids with superior electrochemical properties bring DET into reality. DET of GOx is characterized by the presence of FAD redox peaks in cyclic voltammetry with formal potential close to GOx standard electrode potential, i.e., between −0.4 and −0.5 (Kang et al., 2009). The detection of glucose is then based upon the increase in the anodic FAD peak current in response to glucose oxidation or on decrease in cathodic FAD peak current due to oxygen consumption/reduction. Though glucose sensing using oxygen consumption cannot be considered as third generation sensing but mediator-less sensing at low potential is the key advantage with these sensors.

##### Graphene–CNT hybrid in DET for GOx

**Figure 10:**
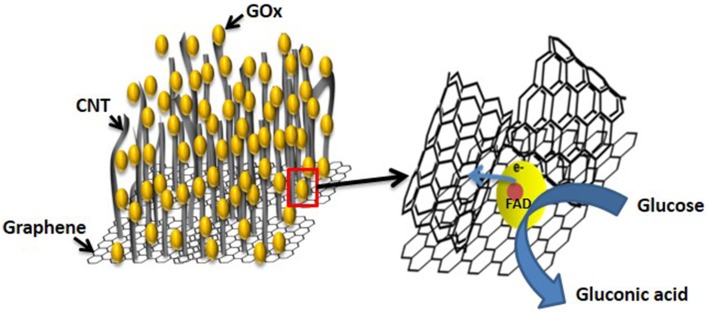
**Schematic representation of electron transfer from redox center of immobilized GOx molecule on G-CNT and enzyme catalyzed oxidation of glucose in biosensing (Not to the scale)**.

G-CNT hybrid provides an increase in surface area for enzyme immobilization and also creates 3D conductive network for efficient electron transfer surrounding enzyme molecules. Figure 10 shows the schematic of GOx immobilized on the G-CNT electrode and DET from enzyme redox center during glucose oxidation. Physically assembled G-CNT hybrid using GO and CNTs has been reported in achieving DET for GOx and glucose biosensing is based on oxygen consumption at cathode (Chen et al., 2012; Mani et al., 2013; Palanisamy et al., 2014). Further, it was reported that G-CNT hybrid modified with ZnO nanoparticles increases the electrochemically active area beneficial for the electron transfer and enzyme immobilization (Hwa and Subramani, 2014). In their approach, differential pulse voltammetry method was used for detection of glucose based on the decrease in oxygen.

##### Graphene-SNMs in DET for GOx

SNMs such as NPs of NiO_2_, TiO_2_, and ZrO_2_ have also been integrated with graphene in electrochemical sensing of glucose (Salimi et al., 2007; Erdem et al., 2014; Hwa and Subramani, 2014; Vilian et al., 2014) due to their ease of preparation, good mechanical, thermal, electrochemical activities, and biocompatible environment to the enzyme. NiO_2_ has a very high pI, it carries a net positive charge at physiological pH which is beneficial for electrostatic immobilization of negatively charged enzymes. TiO_2_ has superior conductivity for faster electron transfer. Composite material of NiO_2_/TiO_2_ with GO, fabricated by layer by layer assembly has been used in DET based glucose biosensing (Xu et al., 2012). ZrO_2_, a transitional metal oxide has low toxicity and is also chemically inert. ZrO2 nanoparticles were electrochemically deposited on the rGO using poly(l-lysine) (PLL) polymer as a linker. Cadmium sulfide (CdS) in the form of nanocrystals, hollow nanospheres, and nanoparticles have been successfully shown to achieve DET for the redox enzyme (Huang et al., 2005; Dai et al., 2008; Wang et al., 2011a).

##### Graphene-MNP in DET for GOx

Graphene structures modified with metal nanoparticles are indispensable in DET and electrochemical sensing. They improve the surface area and provide a conductive and reactive interface for faster electron kinetics. AuNP provide a native redox environment to the proteins giving them more degree of freedom (Zheng et al., 2011). For example, graphene nanosheets modified with AuNP (Chen et al., 2011b; Zhang et al., 2012; Li et al., 2013b; Wang and Zhang, 2013; Xu et al., 2014) as well as G-CNT modified with AuNP (Nayak et al., 2014) have been applied in DET of GOx.

AuNP embedded in a matrix of conducting polymer such as polyaniline (PANI)/graphene together yields a hybrid possessing electrochemical properties of metal and graphene and easy processibility of polymer. PANI provides compatible supporting matrices for both enzyme and AuNP (Zhao et al., 2009; Xu et al., 2010).

Table 1 summarizes performances of various reported graphene hybrids in DET based glucose biosensing.

**Table 1:** **Summary of performances of graphene hybrids in DET based glucose biosensors**.

**Type of Hybrid**	**Electrode**	**Ks (s^−1^)**	**Detection range (mM)**	**Sensitivity (symbolμA mM^−1^cm^−2^)**	**LOD (symbolμM)**	**References**
Graphene-CNT or Graphene-CNT/NP	GOx/electrochemically reduced G-MWCNT/GCE with FMCA	3.02	0.01–6.5	7.95	4.7	Mani et al., 2013
	GOx/chemically reduced G-CNT/GCE	Not reported	Up to 8	1.27	Not reported	Chen et al., 2012
	GOx/MWCNT/GO	11.22	0.1–19.82	0.266	28	Palanisamy et al., 2014
	GOx/graphene/CNT/ZnO	5.544	0.01–6.5	5.362 ± 0.072	4.5 ± 0.08	Hwa and Subramani, 2014
Graphene–SNMs	GOx/CdS nanoparticles	Not reported	0.05–11	7	50	Huang et al., 2005
	GOx/CdS nanocrystals	5.9	2–16	1.76	700	Wang et al., 2011a
	GOx/PLL/RGO/ZrO_2_	5.03 ± 0.14	0.29–14	11.65 ± 0.17	130 ± 21	Vilian et al., 2014
	GOx/NiO_2_/TiO_2_/Graphene	Not reported	1–12	4.129	1.2	Xu et al., 2012
Graphene–metal nanoparticle	GOx-AuNP-chitosan-graphene nanosheets	Not reported	0.0021–0.0057	79.71	0.7	Zhang et al., 2012
	GOx/PAMAM dendrimer/AgNP/RGO	8.59	0.032–1.89	75.72	4.5	Luo et al., 2012
	GOx/AuNP/bilayer graphene	7.74 ± 0.16	0.1–10	For Human serum:64 μA mM^−1^	35	Chen et al., 2011b
	GOx/AuNP/Sulfonated graphene	Not reported	2–16	14.55	200	Li et al., 2013b
	GOx/PANI/AuNP/Graphene	4.8	0.004–1.12	Not reported	0.6	Xu et al., 2014

*Ks, Electron transfer constant; LOD, Limit of detection*.

There are also reports on applications of graphene hybrids in DET of heme peptide at G-CNT hybrid (Komori et al., 2015) and hemoglobin at RGO-CNT (Wang and Zheng, 2012; Sun et al., 2013), GO/AuNP (Xu et al., 2013), and GO/Fe_3_O_4_ NP (Wang et al., 2013c). These DET based electrodes are useful for studying the electron transfer mechanisms of enzymes/proteins as well as for detection of analytes such as hydrogen peroxide (H_2_O_2_) (Wang and Zheng, 2012; Wang et al., 2013a; Komori et al., 2015), trichloroacetic acid (Sun et al., 2013), NaNO_2_(Sun et al., 2013), and nitric oxide (NO) (Xu et al., 2013).

Graphene hybrids have also been used in non-DET based detection of various other targets like small molecules, metabolites, DNA, antibodies, and biomarker. The recent reports in the literature on detection of these targets has been summarized in Table 2.

**Table 2:** **Graphene hybrids used in electrochemical sensors**.

**Graphene hybrid electrode**	**Target**	**Electrochemical method of detection**	**References**
PtAu/RGO/GCE	H_2_O_2_	Chronoamperomety	Cui et al., 2015
PtAu/G-CNTs/GCE			Lu et al., 2013
AuNPs/Graphene/Chitosan/GCE			Jia et al., 2014
AgNPs/MWCNT/rGO			Lorestani et al., 2015
RGO/sandwich-like periodic mesopourous silica (PMS)/AuNPs	Live cancer cells detection (HeLa and HepG2) through H_2_O_2_ sensing, glucose		Maji et al., 2014
Pt/rGO–CNT paper	Real time tracking of H_2_O_2_ secretion by live cells macrophages		Yimin et al., 2015
Cholesterol oxidase/Chloesterol esterase/PtNP/RGO sheets	Cholesterol		Dey and Raj, 2010
Lactate oxidase/TiO_2_/Photocatalytically RGO/GCE	Lactic acid		Casero et al., 2014
Au/(Polypyrrole)PPy/RGO sheets	Dopamine	Differential pulse voltammety	Tao et al., 2014
Graphene/mesoporous silica/AuNPs	DNA		Du et al., 2011b
Aptamer/Graphene/mesoporous silica/Au NPs and silver macrospheres	ATP		Guo et al., 2011
Anti-AFP/AuAg/RGO/Thionine/GCE	Alpha-fetoprotein (AFP)		Su et al., 2011
HIgG Ab/rGO/MWCNT/PdNP	Human IgG (HIgG)	Square wave voltammetry	Liu et al., 2015

#### Graphene hybrids as photochemical/optical sensors

Hybrids using graphene and SNM with their enhanced electronic properties have high potential for use as transducers to transform different forms of energy (solar or chemical) into electrical/optical signals for sensing. The large surface area and adsorptivity of SNM increases the electric response of sensors while graphene enhances adsorption of biomolecules and increases signal-to-noise ratio.

ZnO grown on graphene/PMMA has been used to sense H_2_, wherein the resistance changes with varying H_2_ concentrations (Liu et al., 2013). A graphene top electrode on a layer of vertically aligned ZnO nanowires was used to detect ethanol vapor with the sensitivity of as high as ~9 for 10 ppm ethanol (Yi et al., 2011). Similarly, vertically aligned ZnO nanorods grown on chemically converted graphene has been used in sensing of H_2_S in oxygen at room temperature (Cuong et al., 2010).

The photoactivity of some SNM like QD and TiO_2_ has been used in designing sensors that work on the mechanisms of photoswitching. TiO_2_ photoactivity was used for oxygen sensing by fabricating reproducible photoswitching devices based on TiO_2_-decorated single layer graphene (SLG) (Wang et al., 2011b). Upon UV irradiation, the conductance of the materials decreases due to the photoexcited electrons at the interface that behave as Coulomb traps, quenching the p-type carriers that flow through graphene. After switching off the UV light, the initial conductance of the hybrid is restored. Graphene itself and the graphene/TiO_2_ hybrid don't show electrical response to oxygen under dark conditions. However, under UV irradiation, the hybrid showed a fast linear sensitivity toward oxygen concentration, and a partial restoration of the material's conductance, in the full range of 5–100% with a detection limit of 0.01% O_2_ and signal-to-noise ratio of 3.65 for 5% oxygen. Given that no effects of UV were observed on the conductance of graphene itself and that TiO_2_ film was insulating due to discontinuity of particles, the response is attributed to the synergetic effect of the photoactivity of TiO_2_ and the ultrasensitivity of graphene toward environment effects.

**Figure 11:**
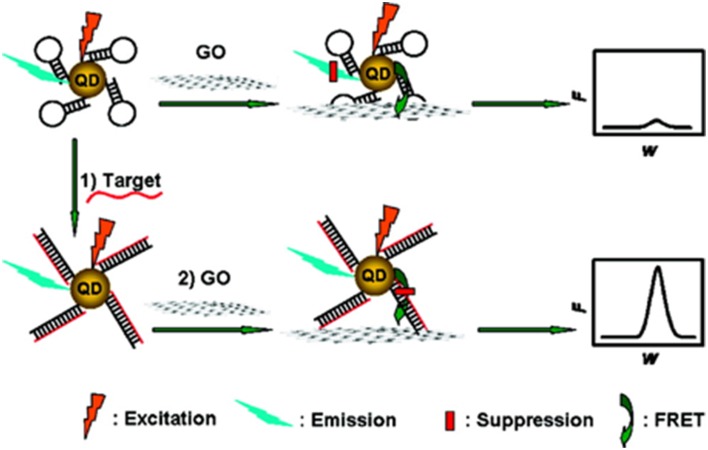
**Biosensing mechanism using GO-induced fluorescence quenching of QDs functionalized with molecular beacons**. In the absence of the target, the fluorescence of the QD is quenched through resonance transfer to the GO. In the presence of the target, resonance transfer is suppressed and the fluorescence signal is high (Dong et al., 2010a). [Reprinted with permission from Dong et al. (2010a). Copyright (2010) American Chemical Society].

Graphene/QDs hybrids have been applied for “turn on” fluorescent biosensors, to detect environmental pollutants. QD are good candidates for energy donors, due to their high quantum yield, narrow, stable fluorescence, and size-dependent tunable absorption and emission, while graphene is the best energy acceptor with high quenching efficiency. The most common technique to combine graphene/QD hybrids as fluorescent biosensors is using aptamers, oligonucleic acids, or peptide molecules that bind to a specific target molecule. Li et al. (2013a) used aptamer-functionalized QDs to bind to graphene sheets enabling the fluorescence quenching due to the energy resonant transfer between the materials to detect Pb(II). When Pb(II) ions are present in the assay the QDs are detached from the GO and the fluorescence turns on. The limit of detection was found to be 90 pM with good selectivity toward Pb(II) in a wide range of metal ions. Dong et al. (2010a) has applied the same principle by using molecular beacon-functionalized QDs (see Figure 11). A strong interaction between the molecular beacon and GO led to fluorescent quenching of the QDs. Upon recognition of the target, the distance between QDs and GO increased, and the fluorescence of the QD increased.

Graphene hybrids have also been studied in detection of metabolites, pathogen/cells, DNA, chemical compounds etc. based on the photochemical/optical methods for other applications. Most of these methods, which are not mentioned before, are based on electrochemiluminescence (ECL), Surface-enhanced Raman Scattering (SERS), and Surface Plasmon resonance (SPR) phenomenon.

Table 3 summarizes recent literature on different photochemical/optical sensors.

**Table 3:** **Graphene hybrids used in photochemical/optical sensors**.

**Graphene hybrid electrode system**	**Target**	**Photochemical/optical method of detection**	**References**
Aptamer/CdTe and GO	Thrombin and DNA	Fluorescence resonance energy transfer (FRET)	Sun et al., 2014
RGO/MWCNT/AuNP	Dopamine	ECL	Yuan et al., 2014
Folic acid/Cystein/carbon nanodots (C-dots)@Ag core shell NP/poly(allyl amine hydrochloride) (PAH)-functionalized RGO	Cytosensing by targeting Folate receptors on Human cervical cancer cells (HeLa) and human breast cancer cells (MCF-7)	ECL	Wu et al., 2013b
Au nanocrystals (AuNCs)/graphene	H_2_O_2_	ECL	Chen et al., 2011c
Sandwich immunosensors with Ab1/AuNP/RGO sheets/GCE and Ab2/SiO_2_ nanospheres/CdTe/CdS core_small_/shell_thick_ QD	Human IgG	Near-infrared electrochemiluminescence (NIR ECL)	Wang et al., 2012
Graphene/MWCNTs/AuNCs	Phenolic compounds	ECL	Yuan et al., 2013
Ab/CdS/Graphene/FTO	Microcystin-LR	Photoelectrochemical method	Tian et al., 2012
Ab/CdSe/TiO_2_–RGO	Carcinoembryonic antigen		Zeng et al., 2014
Popcorn shaped AuNP/GO	HIV DNA and Bacteria	SERS	Fan et al., 2013
CuNPs/Graphene	Adenosine	SERS	Qiu et al., 2015
GO/PDDA/AgNPs	Folic acid	SERS	Ren et al., 2011
Protein A/Au-GO	Rabit IgG	SPR	Zhang et al., 2013

#### Graphene hybrids as chemiresistive sensors

Meng et al. (2015) recently published an extensive review on graphene hybrids used in chemiresistive gas sensors. Some of the important chemiresistive gas sensors reports using G-MNM hybrids and G-SNM hybrids have been summarized under this section.

##### Graphene-nobel metals hybrids in chemiresitive gas sensors

Hydrogen gas has been detected using noble metal/graphene hybrid at room temperature like. Kaniyoor et al. (2009) reported lowest detection limit of 30 ppm using Pd/graphene hybrid. Shafiei et al. (2009) synthesized Pt/graphene with the lowest detection limit of 50 ppm. Chu et al. (2011) reported the detection range of 6–1000 ppm with lowest detection limit of 6 ppm by use of Pd nanocube/ RGO hybrid. Toxic gases such as NH_3_ and NO_2_ has been reported with Ag/RGO composite with the lowest detection limit of 15 and 0.5 ppm by Phan and Chung (2014) and Tran et al. (2014) respectively.

##### Graphene-metal oxides hybrids in chemiresitive gas sensors

Yi et al. (2011) reported the detection limit of 1 ppm of NO_2_ gas with ZnO/graphene composite. Jiang et al. (2005) sensed benzene at elevated temperature of 260°C using composite of SnO_2_/graphene with the lowest detection value of 5 ppb. Likewise, Meng et al. (2009) synthesized CuO_2_/graphene composite and detected H_2_S at room temperature with 5 ppb as lowest level of detection. Zhou et al. (2013) reported lowest detection concentration of 0.4 ppm for NO_2_ with the use of RGO/Cu_2_O nanowire mesocrystals at room temperature. Huang et al. (2013) synthesized flower like SnO_2_/graphene for detection of NH_3_ and achieved the lowest detection limit of 10 ppm at room temperature. Similarly flower like ZnO/RGO was synthesized by Anand et al. (2014) for sensing H_2_ at 150°C and achieved the lowest detection limit of 10 ppm. Hoa et al. (2013) made use of NiO nanosheet/RGO for detecting NO_2_ at 200°C with detection limit of 1 ppm. Chen et al. (2013) exploited NO_2_ based gas sensor based on Co_3_O_4_/RGO which showed an increased response when compared to bare RGO for NO_2_ detection with 1 ppm as lowest detection limit.

#### Graphene hybrids in sensitized solar cells

Sensitized solar cells (SSCs) have attracted considerable interest since Grätzel's group introduced nanostructured TiO_2_ film into anode electrodes in 1991 (Oregan and Gratzel, 1991) due to their relatively low cost and high efficiency for the photoelectrical conversion of solar energy. SSCs are photoelectrochemical systems, composed of a photoanode, a counter electrode, and an electrolyte. The photoanode is usually a semiconductor, mostly ZnO and TiO_2_, sensitized with photoactive materials, like dyes (DSSC), biomolecules (BSSC), and quantum dots (QSSC), deposited on a conductive transparent surface that conducts photogenerated electrons from the semiconductor into the external circuit.

Graphene in SSC has been applied in both photoanode and counter electrode, playing distinct roles. First, given its extremely high mobility, it can be used as transparent conductive film in the photoanode. As conductive film it would substitute currently used metal oxides, such as FTO and ITO, as it overcomes the disadvantages of the metal oxides, such as cost, sustainability, chemical and mechanical resistance, and higher transmittance in the near-infrared range (Faccio et al., 2011). Second, it can act as electrical bridging additive in the semiconductor layer. Third, graphene quantum dots may be used as the sensitizer, with semiconductor properties so that photons excite electrons from its HOMO to its LUMO. Finally, graphene can be part of the counter electrode, to increase electron transfer and improve redox activity. Table 4 summarizes the performance of different types of graphene hybrids based SSCs reported in literature.

**Table 4:** **Summary of SSC performance using graphene hybrids**.

**Electrode with graphene/SNM hybrid**	***J*_sc_ (mA/cm^2^)**	***V*_oc_(V)**	***FF* (%)**	***n* (%)**	**References**
PA: N719/TiO_2_-Graphene Oxide/FTO	13.1	0.77	71	7.26	He et al., 2013
PA: N3/TiO_2_-rGO/	16.29	0.69	NA	6.97	Yang et al., 2010
PA: N719/ TiO_2_-graphene nanofibers/FTO	16.2	0.71	66	7.6	Madhavan et al., 2012
PA: Graphene QD/TiO_2_/FTO	0.2	0.48	58	NA	Yan et al., 2010
PA: ZnO-graphene QD/Cs_2_CO_3_/Al	0.196	0.99	24	2.33	Son et al., 2012
PA: (TNS-rGO-CdS QD)_10_/FTO	0.92	1	41	0.38	Wang et al., 2013b
CE: CuInS_2_-rGO/FTO	16.63	0.74	51	6.18	Liu et al., 2014
CE: Cu_2_S-rGO-PVA binder/FTO	18.4	0.52	46	4.40	Radich et al., 2011
CE: SWCNT-rGO/FTO	12.81	0.9	76	8.37	Zheng et al., 2013
CE: MWCNT-rGO/FTO	16.05	0.75	62.7	7.55	Velten et al., 2012
CE: TiN-rGO-CNT/FTO	14.0	0.642	46	4.13	Youn et al., 2013
PA: MWCNT-rGO-TiO2/FTO	11.27	0.78	70	6.11	Ming-Yu et al., 2011
CE: rGO-CNT/FTO	11.42	0.77	53	4.66	Li-Hsueh et al., 2013
CE: rGO-CNT/FTO	15.25	0.68	51.05	5.29	Ma et al., 2014
CE: CNT-rGO/graphite paper	12.86	0.78	61.3	6.17	Guang et al., 2011
CE: rGOnanoribbons-CNT/FTO	16.73	0.73	67	8.23	Zhibin et al., 2013
CE: VACNT-graphene paper	14.24	0.68	62.4	6.05	Li et al., 2011
CE: Pt NPs/graphene nanosheets/FTO	18.26	0.72	65	8.54	Tsai et al., 2015
CE: graphene nanoplateletes-Pt NPs/FTO	14.31	0.735	61.9	6.51	Hoshi et al., 2014

*PA, Photoanode; CE, Counter electrode; J_sc_, Short circuit current density; V_oc_, Open circuit voltage; FF, Filling factor; n, Energy conversion efficiency*.

##### Graphene-SNM hybrids in SSCs

These hybrids have been used in the photoanode, where graphene acts as a bridge between semiconducting nanoparticles. As a 2D material graphene has intermolecular forces, such as physisorption, electrostatic binding, or charge transfer interactions with TiO_2_ NPs to anchor firmly on the surface and this improved contact allows faster extraction of photoinduced electrons. The graphene/TiO2 interface represents an electronic contact between semiconductors of zero-bandgap and wide bandgap that produces deep depletion of charge carriers with substantial band bending. This inhibits the possibility of back injection, making charge flow unidirectional. Unidirectional flow suppresses recombination and back electrons, main detriments for cell efficiency. Graphene also provides porosity, improving light scattering, which was shown to increase the cell efficiency and short circuit current (Yang et al., 2010). The same study reported that the best performance was obtained with just 0.6% weight load of GO. Graphene additives have proven better than CNT due to the larger extent of interactions and better alignment of energy levels. Madhavan et al. (2012) used mats of TiO_2_/graphene composite nanofibers fabricated by electrospinning on FTO as photoanodes in DSSC, attaining power conversion efficiency of 7.6%. When compared with bare TiO_2_ mats, the V_oc_ was very similar in the two cells, but a significant improvement was found in current density, resulting in an efficiency improvement of 21%.

A stacked structure of titania nanosheets (TNS), rGO and CdS QD has also confirmed the role of graphene in providing additional paths for electrons to be collected in the photoanode and a faster hole transport to the electrolyte, greatly suppressing electron-hole recombination (Yang et al., 2012; Wang et al., 2013b). Titania acts as the antenna pigment to harvest light and excite the electrons while graphene performs as electron acceptor by capturing and transporting to outer circuit. The limitation of titania to UV light absorption is overcome with the larger visible light absorption of QD. A fast and uniform photoresponse was obtained for the QD/TNS/rGO, while negligible response resulted in the absence of QD, reaffirming the role of QD as sensitizers. With the addition of graphene, the efficiency is greatly improved, since graphene could capture and shuttle electrons quickly. A low efficiency of 0.38% can be attributed to the very small thickness of the photoanode, tens of nm, in comparison to common dye-sensitized solar cells that are about 10 μm thick.

**Figure 12:**
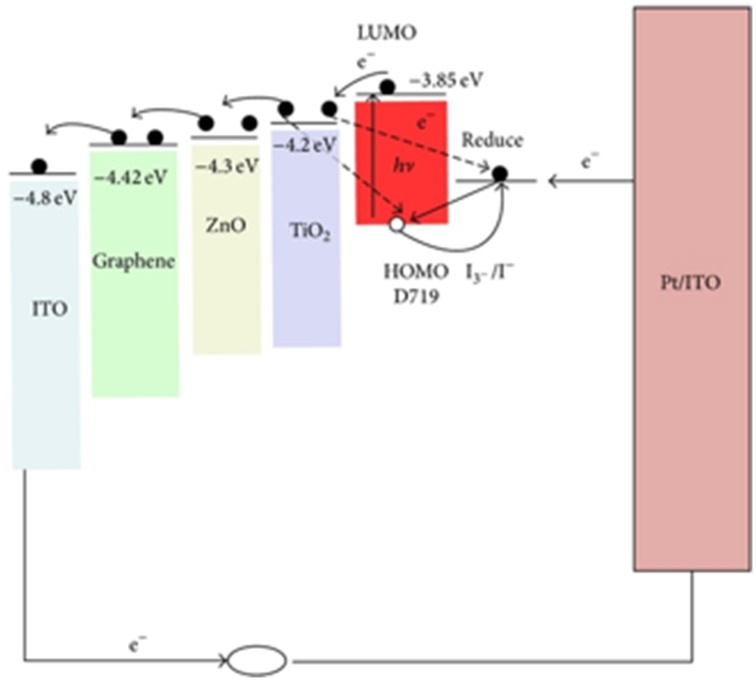
**Energy diagram illustrating the energetically favorable transfer routes for photogenerated charges in hybrid materials at the photoanode (solid arrows) and the undesired recombination processes (dashed arrows) (Chih-Hung et al., 2014)**.

Son et al. (2012) demonstrated ZnO/graphene quasi-core-shell QD sandwich between organic layers to favor efficient dissociation of photogenerated electron-hole pairs. Efficient charge transfer from ZnO conduction band into induced LUMO of graphene enhances the photovoltaic effect of the material. ZnO/graphene quasi-core-shell QD were fabricated by covering ZnO nanoparticles with graphene nanoshells. A solar cell fabricated with the hybrid as sensitizer confirmed that photoinduced charge separation and transport to collecting electrode was improved through static quenching. Due to Zn-O-C bond, the electrons that are photo-excited into the conduction band of ZnO are rapidly transferred into graphene's LUMO and the excited state of ZnO is quickly deactivated making it available for new photoelectric events to occur. Similar energetically favorable charge transfer routes from the conduction band of the semiconductor into graphene LUMO is proposed by Chih-Hung et al. (2014), as illustrated in Figure 12. This way the charge generation efficiency of the hybrid improves in comparison to solely ZnO.

Graphene/SNMs also have been proven advantageous when used as counter electrode. Pt is a well-known counter electrode material due to its high electrocatalytic activity toward the most common electrolyte in SSC, iodide/triiodide. However, due to its scarcity and cost, finding inexpensive substitutes is crucial. A combination of highly electrocatalytic chalcogenide semiconductors and graphene on FTO have proven to be feasible substitutes for Pt (Liu et al., 2014). The resultant efficiency using graphene/CuInS_2_/FTO counter electrode was 6.18%, only slightly lower than the 6.52% of a Pt counter electrode. Graphene's large surface area readily captures the electrons from redox couples in the electrolyte thereby easily reducing them. So, similar to its role as bridge as in the photoanode, the graphene facilitates electron transmission across the counter electrode/FTO interface. However, graphene absorbs light, therefore less light is reflected from the counter electrode and light harvesting efficiency is reduced in comparison to Pt. rGO/Cu_2_S on FTO has also been used as counter electrode for CdS/CdSe/ZnS QSSC by Radich et al. (2011) and Santra and Kamat (2012) with improved cell performance, since Cu_2_S exhibits higher photocatalytic activity toward polysulfide electrolyte reduction than Pt, and rGO platform stabilizes the nanoparticles, its large surface area promotes good dispersion and provides high number of reactive sites, while the good mobility serves to rapidly shuttle electrons to the Cu_2_S catalyst sites. The fill factor of the hybrid was increased in 75% in comparison to Pt, confirming the more efficient shuttling of electrons, resulting in one of the highest power conversion efficiency reported for liquid junction QDSSC.

##### Graphene-CNT hybrids in SSCs

Graphene-CNT hybrid materials have recently been studied for their use mainly as a counter electrode in SSC, but also as part of the photoanode and the electrolyte. Guang et al. (2011) incorporated rGO-CNT in the counter electrode, and observed that CNTs bridged gaps between flakes and improved the electrical conductivity. The good catalytic and electrical properties of the hybrid allowed obtaining a cell performance that was slightly lower but competitive with one using a Pt counter electrode, considering the advantages of cost and mechanical flexibility that carbon nanomaterials provide. Zhibin et al. (2013) demonstrated a higher performance of graphene nanoribbons-CNT hybrid compared to Pt, by partially unzipping MWCNT, which resulted in bridged CNT with very large surface area. Another form of hybrid formed by VACNT grown on graphene paper was developed for SSC counter electrode by Li et al. (2011) allowing the cell efficiency to reach 83% of that with a Pt film electrode and a superior efficiency to tangled CNT due to shorter transportation paths. The benefits of combining MWCNT and graphene in the photoanode have been reported by Ming-Yu et al. (2011), where the adsorption of MWCNTs showed to lessen the recombination and the aggregation between graphene sheets and increased the degree of dye adsorption. Meanwhile, the combination of graphene and CNT with ionic liquids in a quasi-solid state electrolyte has been shown Ahmad et al. (2011) to significantly increase the energy conversion efficiency of the cell from 0.16 up to 2.5%, because the carbon nanomaterials act as charge transporters in the ionic liquid and as catalysts for the electrochemical reduction of I^3−^.

##### Graphene-MNM hybrids in SSCs

The main hybrid under this category is graphene-Pt, since Pt is the standard electrocatalyst for the reduction of I^−3^. The performance of Pt as counter electrode is hard to overlook, however, its high cost is a motivation to search for substitutes. Different architectures of the hybrid have been reported for this application that don't affect the performance of the cell significantly. Tripathi et al. (2014) studied the stacking of a Pt thin film on graphene film and vice versa. Hoshi et al. (2014) reported on preparing a paste of graphene nanosheets-Pt NPs and coat in on a conductive glass. The role of graphene depends on the location in the electrode, where it could act as an improver of electrical contact between Pt and FTO or as a co-catalyst. Bajpai et al. (2012) has reported the alternative use of Ni NPs uniformly anchored on graphene as a Pt-free counter electrode, which in fact yielded a higher efficiency than the standard Pt electrode. The use of graphene-MNM hybrids in the counter electrode can result in reduced manufacturing costs and up-scale the use of SSCs, while opening possibilities for flexible devices.

## Conclusions and future outlook

Whether as a transducer in mediatorless glucose biosensors, a photoactive material, electronic transfer aid, or charge collector platform, graphene's use has been steadily increasing and impacting modern day electronics. Hybridization of graphene with carbon nanomaterials and semiconductors can be performed by innumerable methods that continue to be discovered. Despite its excellent properties, graphene still has a long way to go before it can replace silicon or tin oxide films, largely because it's reproducibility and large scale production with process control of layer number still remain a challenge. When designing graphene hybrid materials great attention must be paid toward optimizing the intrinsic properties of the components simultaneously with the interface structure and mechanisms of electronic coupling, some of which are still unknown. As we have selectively reviewed the recent advances of graphene-CNT, graphene-SNM, and graphene-MNM hybrids with focus on sensing and energy conversion, it can be easily realized that this trend of using graphene hybrids with their multifunctionalities and multimodalities will continue to proliferate in fields of flexible electronics, medical diagnostics, chemical sensing, solar cells, and many other transduction devices.

### Conflict of interest statement

The authors declare that the research was conducted in the absence of any commercial or financial relationships that could be construed as a potential conflict of interest.
